# Deciphering the Prognostic and Predictive Value of Urinary CXCL10 in Kidney Recipients With BK Virus Reactivation

**DOI:** 10.3389/fimmu.2020.604353

**Published:** 2020-12-10

**Authors:** Claire Tinel, Agathe Vermorel, Daniela Picciotto, Lise Morin, Arnaud Devresse, Virginia Sauvaget, Xavier Lebreton, Laïla Aouni, Dominique Prié, Séverine Brabant, Véronique Avettand-Fenoel, Anne Scemla, Marc Olivier Timsit, Renaud Snanoudj, Christophe Legendre, Fabiola Terzi, Marion Rabant, Dany Anglicheau

**Affiliations:** ^1^ Department of Nephrology and Kidney Transplantation, Necker Hospital, Assistance Publique-Hôpitaux de Paris, Paris, France; ^2^ Necker-Enfants Malades Institute, French National Institute of Health and Medical Research, Paris, France; ^3^ Paris University, Paris, France; ^4^ Division of Nephrology, University Hospital Saint-Luc, Brussels, Belgium; ^5^ Institute of Experimental and Clinical Research, Catholic University of Louvain, Brussels, Belgium; ^6^ Department of Physiology, Necker Hospital, Assistance Publique-Hôpitaux de Paris, Paris, France; ^7^ Département of Virology, Necker Hospital, Assistance Publique-Hôpitaux de Paris, Paris, France; ^8^ Department of Urology, Georges Pompidou European Hospital, Assistance Publique-Hôpitaux de Paris, Paris, France; ^9^ Department of Nephrology, Hemodialysis and Kidney Transplantation, Foch Hospital, Suresnes, France; ^10^ Pathology Department, Necker Hospital, Assistance Publique-Hôpitaux de Paris, Paris, France

**Keywords:** urinary chemokines, CXCL10, BK polyomavirus, kidney transplantation, prognostic biomarker

## Abstract

BK virus (BKV) replication increases urinary chemokine C-X-C motif ligand 10 (uCXCL10) levels in kidney transplant recipients (KTRs). Here, we investigated uCXCL10 levels across different stages of BKV replication as a prognostic and predictive marker for functional decline in KTRs after BKV-DNAemia. uCXCL10 was assessed in a cross-sectional study (474 paired urine/blood/biopsy samples and a longitudinal study (1,184 samples from 60 KTRs with BKV-DNAemia). uCXCL10 levels gradually increased with urine (P-value < 0.0001) and blood BKV viral load (P < 0.05) but were similar in the viruria and no BKV groups (P > 0.99). In viremic patients, uCXCL10 at biopsy was associated with graft functional decline [HR = 1.65, 95% CI (1.08–2.51), P = 0.02], irrespective of baseline eGFR, blood viral load, or BKVN diagnosis. uCXL10/cr (threshold: 12.86 ng/mmol) discriminated patients with a low risk of graft function decline from high-risk patients (P = 0.01). In the longitudinal study, the uCXCL10 and BKV-DNAemia trajectories were superimposable. Stratification using the same uCXCL10/cr threshold at first viremia predicted the subsequent inflammatory response, assessed by time-adjusted uCXCL10/cr AUC (P < 0.001), and graft functional decline (P = 0.03). In KTRs, uCXCL10 increases in BKV-DNAemia but not in isolated viruria. uCXCL10/cr is a prognostic biomarker of eGFR decrease, and a 12.86 ng/ml threshold predicts higher inflammatory burdens and poor renal outcomes.

## Introduction

Almost 50 years after its first description in kidney transplant recipients (KTRs) (1), BK polyomavirus (BKV) remains a clinical concern, with BKV-DNAemia still found in 10–30% of KTRs, leading to BKV-associated nephropathy (BKVN) in 1–10% (2). Once it occurs, BKVN represents a serious threat to the renal transplant, causing allograft failure in 15–50% of cases (3). Reducing the detrimental consequences of BKV reactivation on graft outcomes requires close monitoring of the BKV viral load (4). However, although the diagnosis of BKV reactivation is relatively simple, its treatment remains very challenging and still relies on tapering immunosuppression, with a subsequent risk of acute rejection.

Upon development as a biomarker for the noninvasive diagnosis of allograft rejection (5–9), increased levels of urinary chemokines have repeatedly been reported in patients with BKV infection (8, 10–14). Although it has been regarded as a confounding factor to date, we aimed to investigate urinary C-X-C motif chemokine 10 (uCXCL10) as a diagnostic biomarker in the course of BKV infection by determining uCXCL10 levels across different stages of BKV replication, evaluating its potential as a prognostic biomarker in comparison to conventional biological and histological markers, and describing the longitudinal course of uCXCL10 in BKV infection.

## Materials and Methods

### Population

**Figure 1:**
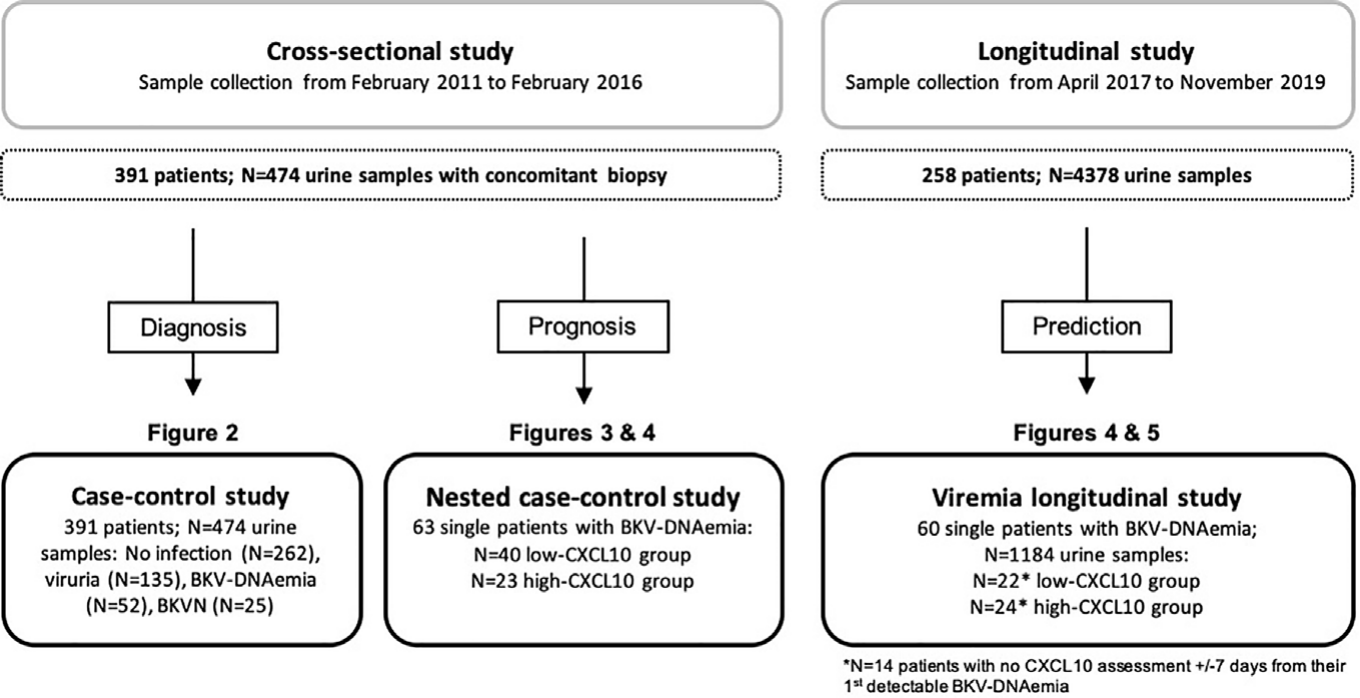
Design of the cross-sectional and longitudinal studies. In the cross-sectional study, all data and samples were collected at a single time point: an allograft biopsy was performed at the time (±7 days) of BKV viral load assessment. The different stages of BKV reactivation were defined as follows: the no BKV infection group, viruria group (viruria was detected in the absence of BKV-DNAemia and BKVN), DNAemia group (positive for BKV-DNAemia, regardless of BKV viruria, with no biopsy-proven BKVN), and BKVN group (positive SV40 staining and/or viral inclusion on the biopsy specimen). Among these 474 samples, BKV-DNAemia was found in N = 52 samples from the DNAemia group and N = 24 samples from the BKVN group. From these 76 samples, we retained the first sample of each patient (N = 63) to participate in the nested case-control study. In the longitudinal study, serial assessment of uCXCL10/cr was performed at predefined time points in all patients consecutively transplanted at Necker Hospital between April 2017 and November 2018. Of these 258 patients, 60 single patients experienced BKV-DNAemia and provided 1,184 samples for uCXCL10/cr quantification. In both BKV-DNAemia cohorts, the patients were divided into 2 groups according to the uCXCL10/cr threshold: the low-CXCL10 group was defined by uCXCL10/cr ≤12.86 ng/mmol, and the high-CXCL10 group was defined by uCXCL10/cr >12.86 ng/mmol.

Cross-Sectional Study (**Figure 1**). We retrospectively considered all adult KTRs followed at Necker Hospital (Paris, France) between February 2011 and February 2016 and selected N = 474 samples collected from N = 391 patients with concomitant (i) blood BKV viral load measurements obtained within ten days before or after allograft biopsy, (ii) informative allograft biopsy, and (iii) available urine samples for research use. Among patients with BKV-DNAemia (N = 76), we retained only the first sample from each patient for the nested case-control study (N = 63).

Longitudinal Study (**Figure 1**). Serial measurement of urinary chemokines became part of routine follow-up during the first year post-transplant at Necker Hospital, starting in April 2017. Urine samples for uCXCL10 quantification were collected at biopsy and at each outpatient clinic visit. We retrospectively considered all consecutive adult patients with at least one positive BKV-DNAemia test from April 2017 on, with a minimum of six months of follow-up (N = 60). Of those 60 individual patients, 46 patients had a urinary chemokine assessment within 7 days before or after their 1^st^ positive BKV-DNAemia. The study was approved by the Ethics Committee of Ile-de-France XI (#13016), and all participating patients provided written informed consent.

### Urine Sample Collection

Urine specimens were collected (immediately before the allograft biopsy if any) and centrifuged at 1,000 x g for 10 min at 4°C within 4 h of collection. The supernatant was collected after centrifugation and stored with (cross-sectional study) or without (longitudinal study) protease inhibitors (cOmplete™, Roche Diagnostics, Meylan, France) at -80°C. Urine cell pellets were resuspended in 1 ml of phosphate-buffered saline and then centrifuged for 5 min at 12,000 x g at room temperature. The supernatant was eliminated, and urine cell pellets were resuspended in RLT Buffer (RNeasy^®^ Mini Kit, Qiagen, Courtaboeuf, France) and stored at -80°C.

### Urine Protein Analyses

Frozen aliquots of urine supernatants were thawed at room temperature immediately before ELISA. Samples were used without dilution and tested in replicate analysis. CXCL10 (Human CXCL10/IP10 Quantikine ELISA kit, Bio-Techne, Minneapolis, USA) was quantified according to the manufacturer’s instructions. Optical densities were derived from a 4-parameter logistic regression of the standard curve. The results were normalized to the urinary creatinine level through determination of the uCXCL10/cr ratio (nanograms of protein per millimole of urinary creatinine).

For the cross-sectional study, ELISA was performed manually, and optical densities were measured using a Multiskan FC plate reader (Thermo Fisher, Illkirch, France). Urine samples with a chemokine concentration below the mean minimum detectable level in the ELISA assay (0.8 pg/ml) were included in the analysis as one-half the detection limit. Measurement of creatinine in urine was performed in the same samples using the Creatinine Parameter Assay Kit (Bio-Techne).

For the longitudinal study, ELISA was performed using an EVOLIS™ Twin Plus System (Clinical Diagnostics, Bio-Rad, Marnes-la-Coquette, France). One-half of the detection limit was 1.95 pg/ml. Measurement of creatinine in urine was performed in the same sample using an Architect c8000 and C16000 (Abbott Diagnostic, Rungis, France).

### BK Virus Analyses

To assess the urine BKV viral load, we used gene-specific oligonucleotide primers and probes (Thermo Fisher) to measure messenger ribonucleic acid (mRNA) encoding BKV VP1 capsid protein as previously reported (15). We used previously published predeveloped and custom-synthetized primers and probes (**Supplemental Table 1**). Total RNA was isolated from urine cell pellets using an RNeasy Mini Kit (Qiagen). RNA concentration was determined using a NanoDrop-2000 spectrophotometer (Thermo Fisher, Montigny le Bretonneux, France). For comparison purposes, RNA samples were concentrated by evaporation for 30 min at 60°C using a SpeedVac™ (Thermo Fisher) and then resuspended at the same concentration of 10 ng/µl in reverse transcription (RT) mix (Taqman Reverse Transcription Reagents, Thermo Fisher). RT was performed on a Veriti^®^ Thermal Cycler (Thermo Fisher) with the following program: 10 min at 25°C, 30 min at 48°C, and then 5 min at 95°C. Then, 1.5 μl of cDNA (after 1/1,000 dilution of RT product for BKV expression) was used for qPCR assay performed in replicate analysis on a Viia™ 7 Real-Time PCR System (Thermo Fisher) using a Fast protocol: 95°C for 20 s, followed by 40 cycles of amplification (95°C for 3 s, 60°C for 30 s). Absolute quantification of gene expression was performed using the murine gene BAK as a standard, with a known number of copies of RNA per µg, with the final result expressed as the number of copies per nanogram of total RNA. A second assay was performed when inconsistent results were obtained (N = 7 cases negative for BKV viruria and positive for BKV-DNAemia).

BKV-DNAemia in whole blood samples was monitored in our hospital laboratory by real-time qPCR (BK Virus R-gene, BioMérieux^®^, Marcy l’Etoile, France) with a positive threshold value of 2.4 Log_10_ copies per ml (500 copies/ml), the lower limit of detection for the assay.

The different stages of BKV reactivation were defined as follows: the no BKV infection group, viruria group (viruria detected with no BKV-DNAemia or BKVN), DNAemia group (positive for BKV-DNAemia, regardless of BKV viruria, in the absence of biopsy-proven BKVN) and BKVN group (positive SV40 staining and/or viral inclusion on biopsy specimen).

### Histology

Biopsy specimens were ﬁxed in formalin, acetic acid and alcohol and embedded in parafﬁn. Tissue sections were stained with hematoxylin and eosin, Masson’s trichrome, periodic acid–Schiff reagent, and Jones stain for light microscopy evaluation. C4d immunohistochemical staining was systematically performed (with rabbit anti-human monoclonal anti-C4d; 1/200 dilution; Clinisciences, Nanterre, France). Clinically indicated or for-cause biopsies were classiﬁed using the 2015 update of the Banff 1997 classiﬁcation (16).

All biopsies performed in patients with concomitant BKV-DNAemia were reviewed by two investigators (MR, AV), and SV40 immunohistochemical staining was systematically performed (anti-SV40 T Antigen Mouse mAb (PAb416), Calbiochem^®^, USA).

### Donor-Specific Antibodies

All circulating donor-specific anti-human leukocyte antigen antibodies (DSAs) were determined with single-antigen flow bead assays (One Lambda, Canoga Park, USA) on a Luminex platform in a single laboratory (Saint-Louis Hospital, Paris). Beads showing a normalized mean fluorescence intensity greater than 500 were considered positive.

### Urinary Tract Infection

Cytobacterial examination of urine was systematically performed at the time of urine collection. Urinary tract infection (UTI) was defined by bacteriuria ≥103 colony-forming units (CFU) and leukocyturia ≥104 white blood cells per ml. Both symptomatic and asymptomatic UTIs were included.

### Statistical Analyses

The results are presented as the mean ± standard deviation (SD) for continuous variables, except for time from transplant to biopsy, viral loads and CXCL10 levels, which are presented as the median and interquartile range [IQR]. The frequencies of categorical variables are presented as numbers and percentages. The distribution of uCXCL10/cr exhibited considerable positive skew, which was substantially reduced by use of natural logarithm (ln) transformation. We compared groups using the Mann-Whitney test or Kruskal-Wallis test followed by Dunn’s post-test, when appropriate. We compared proportions using Fisher’s exact test or a Chi-2 test with Yate’s continuity correction when appropriate. We used a parametric Pearson correlation test on log-transformed variables when the sample size was >30. P-values ≤0.05 were regarded as statistically significant.

To identify parameters independently associated with allograft prognosis after BKV-DNAemia, death-censored Cox regression analysis (**Table 2**) was performed. We tested for biologically and histologically relevant variables for explaining a given decrease in graft function. Natural logarithm transformation was used to reduce right skewness. A multivariate model was built, including all variables with P <0.2 in the univariate analysis. With a 50% eGFR decrease event occurring in 23 patients, a minimum of “10 events per variable” was respected, with no more than three variables entered in the final multivariate regression. The Kaplan-Meier method was used for the survival analyses.

A random forest classification analysis was also used to address the importance of variables in explaining allograft prognosis after BKV-DNAemia. Out-of-bag error (the error rate from samples not used in the construction of a given tree) was minimized by tuning the number of trees (ntrees = 1,000) and the number of variables randomly chosen at each node (mtry = 4). The results are given as a variable importance measure according to the mean Gini decrease.

Finally, to compute the trajectory analyses (**Figures 4A, B** and **Supplemental Figure 3**), the k-nearest neighbors method was used for local regression of the longitudinal data (blood BKV viral load and uCXCL10/cr), followed by modeling of the regression for the entire sample period. The urinary CXCL10/cr area under the curve (AUC) was calculated for each patient from first BKV-DNAemia to BKV negativity, censored by the rejection date, if any. To reflect the intensity of the immune response and not the time to BKV clearance, the AUC was normalized by the number of days of BKV-DNAemia and thereupon expressed as time-adjusted uCXCL10/cr AUC (ng/mmol/d). In this analysis, DNAemia lower than the limit of quantification (LOQ) was included as one-half of the LOQ.

Outcomes were determined as of November 29, 2019, for the cross-sectional cohort and as of April 20, 2020, for the longitudinal cohort.

Analyses were performed with R software (R Development Core Team, R version 3.6.3 and R studio version 1.2.5033) and GraphPad PRISM^®^ Software GraphPad Software, San Diego, USA, version 7.0a).

## Results

### Cross-Sectional Cohort

**Figure 2:**
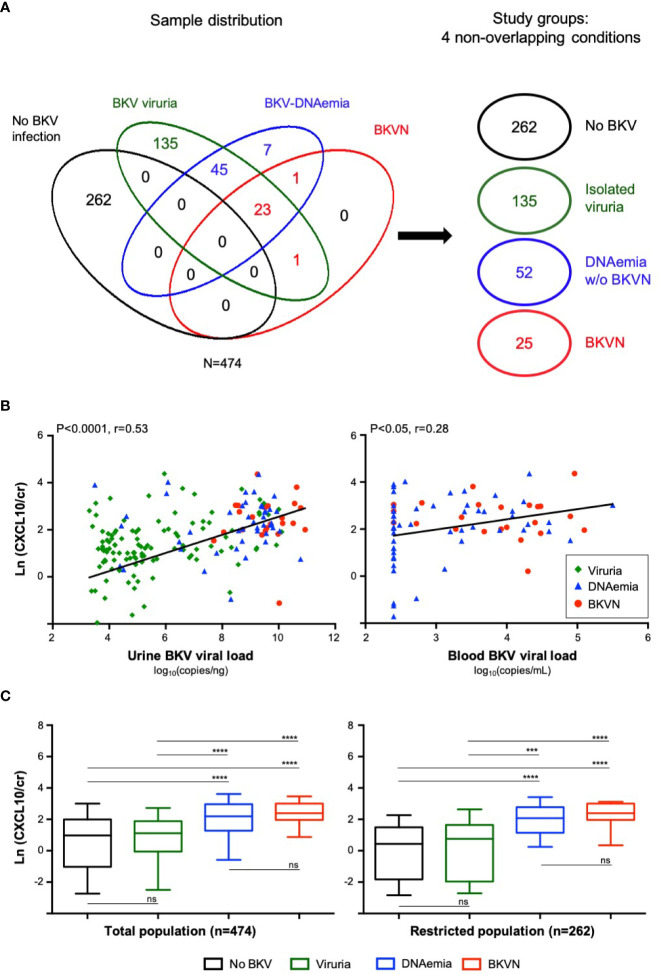
Sample distribution and chemokine levels in the cross-sectional study **(A).** Euler diagram illustrating the sample distribution according to detection of viruria, BKV-DNAemia and histological diagnosis of BKVN and the constitution of four non-overlapping groups according to BKV status: no BKV infection, BKV viruria, BKV-DNAemia without BKVN and BKVN **(B)**. Urine BKV viral load among the different groups (viruria, BKV-DNAemia and BKVN) and its correlation with uCXCL10/cr levels. Similarly, the blood BKV viral load in the BKV-DNAemia and BKVN groups and its correlation with uCXCL10/cr levels is shown. Correlations were computed from Pearson’s test **(C).** Urinary CXCL10 levels according to the 4 BKV groups in the total population and in a restricted population after exclusion of confounding factors. In this restricted population, we excluded samples with significant UTI, with no cytobacterial examination available, with a concurrent acute rejection diagnosis or with inadequate biopsy. P-values were obtained from a Kruskal-Wallis test followed by Dunn’s multiple comparisons ***P < 0.001; ****P < 0.0001. BKVN, BKV-associated nephropathy; Cr, urinary creatinine; Ln, natural logarithm; UTI, urinary tract infection; ns, not significant.

In this study, 474 sets of three samples (i.e., allograft biopsy/urine/blood) collected from 391 individual patients were identified (**Figure 1**). **Supplemental Table 2** shows the patient characteristics at the time of transplantation. Biopsies were performed at a median time of 11 [IQR: 34] months post-transplantation and were mainly clinically indicated (87.8%, **Table 1**). At the time of biopsy, the mean serum creatinine was 185 ± 99 µmol/L. BKV-DNAemia was detectable in 16% of cases, with a median viral load of 3.32 [IQR: 3.1] Log_10_ copies/ml, and 5.3% met the criteria for BKVN (N = 25). BKV viruria was detectable in 43% of cases, with a median viral load of 7.2×10^5^ [IQR: 6.8×10^8^] Log_10_ copies/ng. As expected, BKV viruria was found in most BKV-DNAemia (86.5%) and BKVN samples (96%). To further address whether different stages of BKV infection might impact uCXCL10 levels, we categorized samples into 4 non-overlapping groups (**Figure 2A**) according to their BKV status: the no BKV infection group (N = 262 samples), viruria group (N = 135), DNAemia group (N = 52), and BKVN group (N = 25).

**Table 1:** Sample characteristics from the four non-overlapping groups in the cross-sectional study.

Variables	All samples n = 474	No BKV n = 262	Viruria n = 135	DNAemia n = 52	BKVN n = 25	P-value
Time after transplantation (mo), *median (IQR)*	11 (34)	11 (46)	11 (28)	9 (19)	7 (17)	0.97
**Indication for biopsy**						
Screening biopsy, *n (%)*	58 (12.2)	31 (11.8)	24 (17.8)	3 (5.8)	0	<0.05
Clinically indicated biopsy, *n (%)*	416 (87.8)	231 (88.2)	111 (82.2)	49 (94.2)	25 (100)	
Allograft dysfunction, *n (%)*	259 (62.3)	163 (70.6)	70 (63.1)	19 (38.8)	7 (28.0)	<0.0001
Proteinuria, *n (%)*	39 (9.4)	27 (11.7)	11 (9.9)	0	1 (4.0)	0.06
*De novo* DSAs, *n (%)*	12 (2.9)	7 (3.0)	5 (4.5)	0	0	0.35
BKV DNAemia, *n (%)*	47 (11.3)	3 (1.3)	0	28 (57.1)	16 (64.0)	<0.0001
Other, *n (%)*	59 (14.2)	31 (13.4)	25 (22.5)	2 (4.1)	1 (4.0)	<0.01
**Pathologic primary diagnosis** (except BKVN)						
Inadequate, *n (%)*	32 (6.8)	18 (6.9)	8 (5.9)	6 (11.5)	1 (4.0)	0.52
Acute rejection, *n (%)*	102 (21.5)	65 (24.8)	28 (20.7)	9 (17.3)	1 (4.0)	0.08
Normal, *n (%)*	23 (4.9)	13 (5.0)	7 (5.2)	3 (5.8)	NA	0.97
Other lesions, *n (%)* ^a^	292 (61.6)	166 (63.4)	92 (68.1)	34 (65.4)	NA	0.64
**BKV infection characteristics**						
Detectable BKV DNAemia, *n (%)*	76 (16.0)	NA	NA	52 (100)	24 (96.0)	0.71
Viral load (Log_10_ copies/mL), *median (IQR)*	3.32 (3.1)	NA	NA	2.68 (1.2)	3.96 (1.9)	<0.001
Detectable BKV viruria, *n (%)*	204 (43.0)	NA	135 (100)	45 (86.5)	24 (96.0)	<0.0001
Viral load (copies/ng), *median (IQR)*	7.2E+05 (6.8E+08)	NA	7.3E+04 (2.2E+06)	6.8E+08 (3.2E+09)	3.6E+09 (1.2E+10)	<0.0001
BKVN, *n (%)*	25 (5.3)	NA	NA	NA	25 (100)	NA
**Laboratory test results at the time of biopsy**						
Serum creatinine (μmol/L), *mean ± SD*	185 ± 99	194 ± 116	176 ± 78	167 ± 53	186 ± 71	0.61
DSAs, *n (%)* ^b^	184 (40.5)	105 (42.0)	51 (38.9)	18 (37.5)	10 (40.0)	0.91
Proteinuria/creatininuria ratio (g/g), *mean ± SD*	0.85 ± 1.6	1.0 ± 1.9	0.71 ± 1.4	0.49 ± 0.7	0.34 ± 0.6	<0.05
Bacteriuria (≥ 10^3^/ml) and leukocyturia (≥ 10^4^/ml)^c^, *n (%)*	43 (10.3)	29 (12.6)	6 (5.1)	6 (12.2)	2 (8.7)	0.17

BKVN, BKV-associated nephropathy; DSAs, donor-specific antibodies; IF/TA, interstitial fibrosis/tubular atrophy; IQR, interquartile range; SD, standard deviation. ^a^Including calcineurin inhibitor toxicity, IF/TA, and recurrent disease. ^b^Data not available (NA) for 20 patients. ^c^NA for 55 patients.

### Urine Levels of CXCL10 Are Significantly Correlated With Urine and Blood BKV Viral Load

We next investigated urine BKV viral load and its correlation with uCXCL10/cr levels. As shown in **Figure 2B**, the median level of BKV viruria gradually increased in the viruria (7.3×10^4^ Log_10_ copies/ng), DNAemia (6.8×10^8^ Log_10_ copies/ng), and BKVN (3.6×10^9^ Log_10_ copies/ng) groups and was strongly correlated with uCXCL10/cr levels (P < 0.0001, Pearson r = 0.53). Similarly, the median BKV-DNAemia was higher in the BKVN group than in the BKV-DNAemia group (3.96 vs 2.68 Log_10_ copies/ml, P < 0.001) and also correlated with uCXCL10/cr to a lesser extent (P < 0.05, Pearson r = 0.28, **Figure 2B**, **right Panel**). As previously reported (17), BKV loads in the urine and blood were correlated (P < 0.01, **Supplemental Figure 1A**).

### Urine Levels of CXCL10 Are Similarly Increased in BKV-DNAemia and BKVN but Not in Isolated BKV Viruria

In the whole population (N = 474), uCXCL10/cr (**Figure 2C**, **left Panel**) remained low in the viruric group as in the absence of BKV reactivation (1.12 vs 0.98, P > 0.99). As expected, uCXCL10/cr was significantly increased in patients with BKV-DNAemia with or without biopsy-proven BKVN (both P < 0.0001). Interestingly, there was no significant difference in chemokine levels between the BKV-DNAemia group and the BKVN group (P > 0.99). In a sensitivity analysis, we excluded samples with suspected urinary tract infection (or no cytobacterial examination of the urine, N = 98) and those with concurrent acute rejection (or inadequate biopsy, N = 135), all established confounding factors for increased uCXCL10/cr (14). In this restricted population (N = 262) where the uCXCL10/cr is hypothesized to be only driven by BKV infection, the results remained unchanged (**Figure 2C**, **right panel**).

Finally, we identified 25 patients from the viruric group with a unexpected high uCXCL10 level. Of those, 80% actually have concurrent acute rejection or UTI, and only few remained in the restricted population as illustrated in **Supplemental Figure 1B**. For the 5 remaining cases, we cannot exclude that other confounders were missed or that the intensity of BKV replication within the urinary tract led to a significant inflammatory response.

### Urinary CXCL10 Levels in Patients With BKV-DNAemia Is a Prognostic Marker of Allograft Function

**Figure 3:**
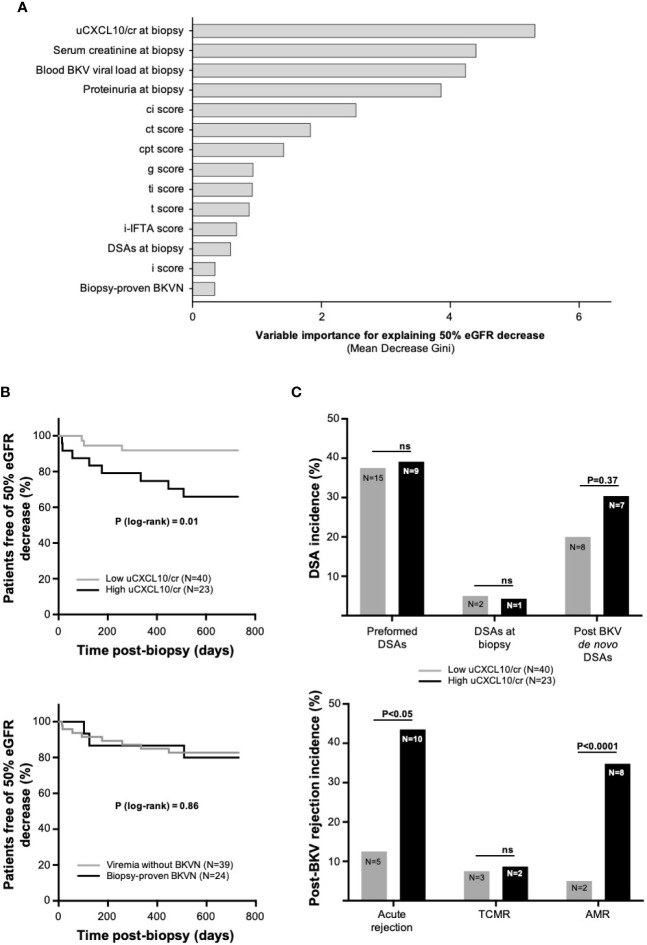
BKV-DNAemia prognosis analysis **(A).** Variable importance measures from a random forest analysis. A total of 1,000 classification trees were built to address the endpoint “50% eGFR decrease” in the 63 patients with BKV-DNAemia (nested case-control study). Fourteen variables were included among the biological and histological data. The mean decrease in Gini is the average of a variable’s total decrease in node impurity, weighted by the proportion of samples reaching that node in each individual decision tree. A higher mean decrease in Gini indicates higher variable importance **(B)**. Kaplan-Meier curves illustrating survival before the occurrence of a 50% eGFR decrease in the low- and high-CXCL10 groups (upper panel) and in two groups of viremic patients with or without BKVN (lower panel). The P-value was computed from a log-rank test **(C)**. Histograms comparing DSA incidence in the low- and high-CXCL10 groups (upper panel) at different time points: preformed DSAs, DSAs at the time of biopsy and post-BKV *de novo* DSAs. The lower panel illustrates the post-BKV occurrence of acute rejection, TCMR and AMR. The P-value was computed from Fisher’s exact test. The low-CXCL10 group was defined by uCXCL10/cr ≤12.86 ng/mmol, and the high-CXCL10 group was defined by uCXCL10/cr >12.86 ng/mmol. AMR, antibody-mediated rejection, BKVN, BKV-associated nephropathy; cr, urinary creatinine; ci, interstitial fibrosis; ct, tubular atrophy; DSAs, donor-specific antibodies; eGFR, estimated glomerular filtration rate; g, glomerulitis; i, interstitial infiltrate; i-IFTA, inflammation within areas of interstitial fibrosis and tubular atrophy; ptc, peritubular capillaritis; t, tubulitis; TCMR, T-cell mediated rejection; ti, total inflammation; ns, not significant.

In the nested case-control study, we focused on renal allograft outcomes among KTRs with BKV-DNAemia, with or without BKVN. We retained only the first sample from each patient, leading to N = 63 unique patients with the set of three samples (i.e., biopsy/urine/blood). **Supplemental Table 3** shows the patient and transplant characteristics. The median time from transplantation to biopsy was 10 [IQR: 19] months, and the median follow-up time was 55 [IQR: 38] months. Nine patients (17%) had a concurrent diagnosis of acute rejection. The determinants, at the time of biopsy, of subsequent allograft function worsening, as assessed by a 50% eGFR decline, were studied by random forest analyses and a Cox proportional hazard model. The random forest analysis (**Figure 3A**) reveals the relative variable importance for explaining the renal outcome (mean decrease in Gini score), allowing visual comparison the respective weight of each explicative variable. As expected, blood viral load, kidney function, and proteinuria were relevant in explaining graft decline. However, uCXCL10/cr clearly outperformed these biological variables as well as the histological diagnosis of biopsy-proven BKVN. Consistently, the multivariate Cox model confirmed uCXCL10/cr as the unique determinant of subsequent graft function decline [HR = 1.52, 95% CI (1.00–2.30), P < 0.05], independent of allograft function (P = 0.46) and blood viral load (P = 0.50) at the time of biopsy or the presence of biopsy-proven BKVN (P = 0.84) (**Table 2**). A threshold of 12.86 ng/mmol discriminated patients with a low risk of postbiopsy graft function decline from the high-risk group (**Figure 3B**, log-rank P-value = 0.01). In contrast, the same survival analysis comparing BKV-DNAemia with and without BKVN showed no difference (log-rank P-value = 0.86).

**Table 2:** Determinants of worsening postbiopsy allograft function, as assessed by the time to reach 50% eGFR decline, by univariate and multivariate death-censored Cox analyses.

Variable category	Explicative variables	Univariate HR (95% CI)	Univariate P-value	Multivariate HR (95% CI)	Multivariate P-value
**Biological data**	Serum creatinine	1.56 (0.45–5.36)	0.4806		
	DSAs at the time of biopsy	0.81 (0.33–2.01)	0.6550		
	Proteinuria/creatininuria ratio	1.60 (0.94–2.73)	**0.0826**	1.55 (0.90–2.68)	0.1153
	Blood BKV viral load	2.88 (0.63–13.13)	**0.1707**	2.01 (0.38–10.66)	0.4142
	Urine BKV viral load	0.96 (0.87–1.06)	0.3983		
**Histological grading**	i Banff elementary lesion	0.57 (0.20–1.57)	0.2744		
	t Banff elementary lesion	1.17 (0.87–1.57)	0.3116		
	ci Banff elementary lesion	1.14 (0.77–1.70)	0.5090		
	ct Banff elementary lesion	1.10 (0.74–1.64)	0.6329		
	ti Banff score	0.93 (0.61–1.42)	0.7528		
	i-IFTA Banff score	1.00 (0.69–1.45)	0.9947		
	MVI score	1.28 (0.44–3.77)	0.6518		
	BKVN	0.98 (0.38–2.49)	0.9610		
**Urinary biomarker**	uCXCL10/cr	1.65 (1.08–2.51)	**0.0193**	1.52 (1.00–2.30)	**0.0473**

BKVN, BKV-associated nephropathy; CI, confidence interval; ci, interstitial fibrosis; cr, urinary creatinine; ct, tubular atrophy; DSAs, donor-specific antibodies; eGFR, estimated glomerular filtration rate; HR, hazard ratio; I, interstitial infiltrate; i-IFTA, inflammation within areas of interstitial fibrosis and tubular atrophy; MVI, microvascular inflammation; t, tubulitis; ti, total inflammation.

Variables with a P-value <0.2 (in bold) in the univariate Cox model were further entered into multivariate (death censored) Cox analysis. MVI is defined by the sum of the glomerulitis and peritubular capillaritis scores.

### Allograft Rejection Drives the Evolution of Renal Function After BKV-DNAemia

As our multivariate analysis identified uCXCL10/cr at the time of biopsy as an independent predictor of postbiopsy graft dysfunction, we aimed to identify the underlying determinants that link uCXCL10/cr at the time of biopsy to postbiopsy graft dysfunction. At the time of biopsy, the patients with low and high uCXCL10/cr levels were similar with regard to eGFR (P = 0.83), BK blood viral load (P = 0.59), peak viral load (P = 0.16), primary histological diagnosis of acute rejection (P = 0.36), and BKVN (P = 0.55, **Supplemental Table 4**).

**Figure 4:**
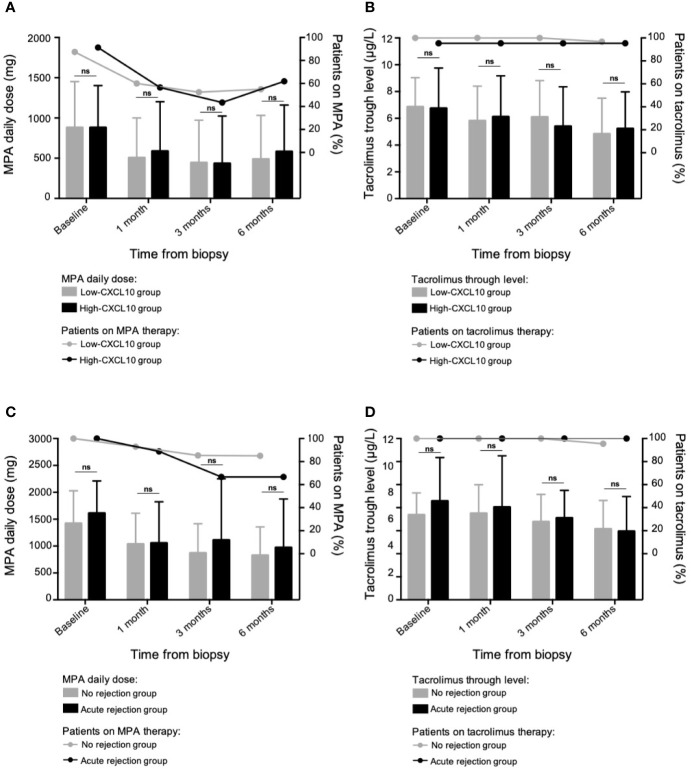
Tapering of the maintenance immunosuppressive regimen. In the case-control study (63 patients with BKV-DNAemia), evolution of the mycophenolic acid daily dose **(A)** and the tacrolimus trough levels **(B)** at different time points following BKV-associated biopsy according to the uCXCL10/cr group. The low-CXCL10 group was defined by uCXCL10/cr ≤12.86 ng/mmol, and the high-CXCL10 group was defined by uCXCL10/cr >12.86 ng/mmol. In the longitudinal study (60 patients with BKV-DNAemia), evolution of the mycophenolic acid daily dose **(C)** and the tacrolimus trough levels **(D)** at different time points following the 1^st^ positive BKV viral load and according to the occurrence of subsequent rejection. Data are presented as the mean ± SD values. P-values were obtained *via* the Mann–Whitney test. The proportions of patients on or off each treatment were compared using Fisher’s exact test in Panels **(A–D)** without any significant differences. MPA, mycophenolic acid; ns, not significant; SD, standard deviation.

BKV-DNAemia led to tapering of the immunosuppressive regimen, with no significant difference between the two groups with regard to mycophenolic acid daily dose or tacrolimus trough levels at baseline, 1–3 and 6 months after biopsy (**Figures 4A, B**).

*De novo* DSAs occurred in 30.4% of patients in the high-CXCL10 group compared to 20% of those in the low-CXCL10 group, but this difference did not reach significance (P = 0.37, **Figure 3C**). However, within a median time of 6 months postbiopsy, acute rejection occurred significantly more often in the high-CXCL10 group than in the low-CXCL10 group (P < 0.05), consisting of mainly AMR (34.8 vs 5%, P < 0.0001, **Figure 3C**). Of note, baseline ABMR frequency was similar between both groups (P = 0.25). Most importantly, 80% of rejection cases occur *de novo* with only 3 recurrent/persistent rejections (**Supplemental Figure 2**). Altogether, this information suggests that clinical prognosis relies on uCXCL10 and/or subsequent ABMR rather than baseline concurrent rejection.

### Longitudinal Cohort: Validation of uCXCL10 Cut-Off as a Prognostic and Predictive Biomarker

Starting in April 2017, serial measurement of uCXCL10 became part of routine follow-up during the first year post-transplantation at Necker Hospital. We retrospectively considered all consecutive adult patients with at least one positive BKV-DNAemia test from April 2017 on, with a minimum of six months of follow-up. In this longitudinal cohort, 60 patients experienced BKV-DNAemia, within a median time of 5 [IQR: 7.3] months after transplantation.

**Figure 5:**
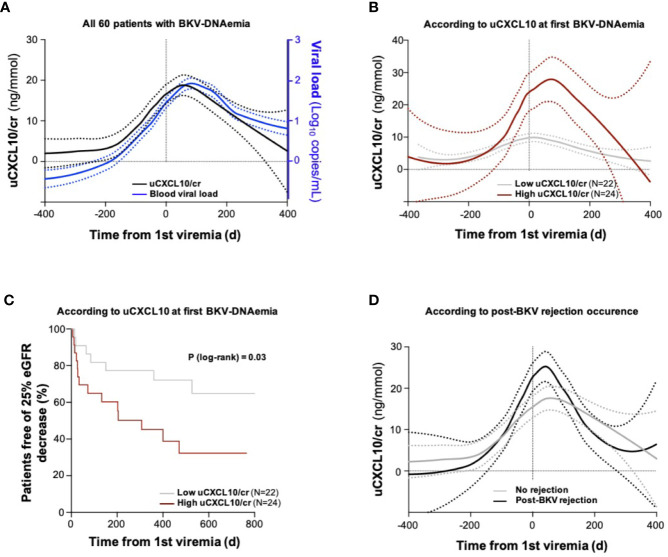
Longitudinal study of urinary CXCL10 in BKV viremic patients **(A).** Urinary CXCL10/cr and blood BKV viral load trajectory analyses in the longitudinal cohort including 60 single patients with BKV-DNAemia. Trajectories were computed by regression from longitudinal assessments of uCXCL10/cr (samples collected at biopsy and each outpatient clinic visit during the 1^st^ year post-transplantation, black line) and all available blood BKV viral loads over the same period (blue line). Dotted lines indicate the confidence interval of each group **(B)**. Urinary CXCL10/cr trajectory according to uCXCL10/cr threshold at first BKV-DNAemia. The low-CXCL10 group (gray line) is defined by uCXCL10/cr ≤12.86 ng/mmol, and the high-CXCL10 group is defined by uCXCL10/cr >12.86 ng/mmol (burgundy line) **(C)**. Kaplan-Meier curves illustrating survival before the occurrence of a 25% eGFR decrease in the low-CXCL10 (gray curve) and high-CXCL10 (burgundy curve) groups. The P-value was computed from a log-rank test **(D)**. Patients were divided according to the occurrence of a post-BKV acute rejection episode (black line) or not (gray line). Dotted lines indicate the confidence interval of each group. cr, urine creatinine; d, days; eGFR, estimated glomerular filtration rate.

The patient and transplant characteristics are shown in **Supplemental Table 5**. Using regression analyses, we computed the longitudinal trajectories of blood BKV viral load and uCXCL10/cr quantified in 1,184 urine samples from these 60 patients. As depicted in **Figure 5A**, the course of uCXCL10/cr parallels that of BKV-DNAemia throughout infection, with concomitant onset and resolution. Moreover, the longitudinal cohort was split in three groups, according to the latest guidelines (4). As suggested on **Supplemental Figure 3**, uCXCL10/cr gradually increased among transient, sustained low-level and sustained high-level DNAemia groups. However, a larger sample size would be needed to reach significance in predicting the subsequent DNAemia profile, *i.e.* in identifying from the very first viremia, KTRs who could benefit from lowering of maintenance immunosuppression.

Next, we sought to validate the relevance of the uCXCL10/cr threshold (12.86 ng/mmol) established in the nest case-control cohort as a prognostic biomarker for allograft function. The longitudinal cohort was split into two groups according to uCXCL10/cr levels at the 1^st^ BKV-DNAemia (≤ or >12.86 ng/mmol). The low- and high-CXCL10 groups had similar characteristics at first BKV-DNAemia, regarding median time post-transplantation (P = 0.24), BKV viral load (P = 0.25), and eGFR (P = 0.18) (**Table 3**). In contrast, the uCXCL10/cr threshold at the first BKV-DNAemia identified two distinct populations regarding the outcome of the urinary inflammatory response (**Figure 5B**). Patients with uCXCL10/cr ≤12.86 ng/mmol at first BKV-DNAemia had consistently low uCXCL10/cr levels throughout the follow-up period. In contrast, patients with uCXCL10/cr >12.86 ng/mmol at first BKV-DNAemia experienced a sharp peak in uCXCL10/cr [36.0 (31.2) vs 12.3 (13.4) ng/mmol, P < 0.0001]. To quantify the urinary inflammatory burden, we computed the area under the uCXCL10/cr curve (AUC) during BKV infection, normalized by the number of days. The time-adjusted uCXCL10 AUC was 19.3 (20.1) in the high-CXCL10 group compared to 7.15 (6.9) ng/mmol/d in the low-CXCL10 group (P < 0.001). These two inflammatory patterns were not associated with BKV clearance time [132 (148) vs 131 (205) days, P = 0.49] or peak BKV viral load [3.6 (1.3) vs 3.6 (1.2), P = 0.89].

**Table 3:** BKV infection characteristics in the longitudinal study, according to urinary CXCL10 at first DNAemia.

According to urinary CXCL10 at first DNAemia				
Variables	All patients N = 60*	Low CXCL10 N = 22	High CXCL10 N = 24	P value
**Characteristics at first DNAemia**				
Time from transplantation (mo), *median (IQR)*	5.0 (7.3)	4.8 (7.5)	4.1 (3.7)	0.24
eGFR (MDRD) (ml/min), *median (IQR)*	44.2 (21.7)	46.7 (17.2)	41.5 (24.6)	0.18
BKV viral load (Log_10_ copies/ml), *median (IQR)*	3.0 (0.8)	2.8 (0.5)	3.2 (0.8)	0.25
Urinary CXCL10 (ng/mmol), *median (IQR)*	13.6 (11.1)	7.6 (5.7)	18.9 (9.6)	<0.0001
**Evolution during DNAemia**				
Peak BKV viral load (Log_10_ copies/ml), *median (IQR)*	3.55 (1.3)	3.6 (1.2)	3.6 (1.3)	0.89
BKVN, *n (%)*	8 (13.3)	3 (13.6)	4 (16.7)	1
BKV clearance time (d), *median (IQR)*	105 (146)	131 (205)	132 (148)	0.49
Peak urinary CXCL10, *median (IQR)*	20.2 (26.9)	12.3 (13.4)	36.0 (31.2)	<0.0001
Urinary CXCL10 daily AUC during DNAemia (ng/mmol/d), *median (IQR)*	10.3 (13.3)	7.15 (6.9)	19.3 (20.1)	<0.001

AUC, area under the curve; BKVN, BKV-associated nephropathy; d, days; eGFR, estimated glomerular filtration rate; IQR, interquartile range; LOQ, limit of quantification; MDRD, modification of diet in renal disease; mo, months; qPCR, quantitative polymerase chain reaction; SD, standard deviation.

CXCL10 groups are defined according to uCXCL10/cr at first DNAemia: ≤12.86 ng/mmol (low) or >12.86 ng/mmol (high). First DNAemia is defined by the date of the first blood BKV qPCR ≥ LOQ. BKV clearance is defined by the time between first DNAemia and the 1^st^ qPCR <LOQ with 2 consecutive concordant assessments. Urinary CXCL10 time-adjusted AUC (ng/mmol/d) is calculated from first DNAemia to BKV clearance, normalized by the number of days of BKV-DNAemia, and censored by the rejection date if any. *N = 14 patients with no urinary CXCL10 assessments within 7 days from their 1^st^ DNAemia could not be classified into the low/high-uCXCL10 groups.

Besides, the same uCXCL10/cr threshold discriminated patients with a low risk of 25% eGFR decrease from high-risk patients (**Figure 5C**, log-rank P-value = 0.03). In a Cox proportional hazards regression model including uCXCL10/cr, eGFR and BKV viral load (all measured at the time of first BKV-DNAemia), uCXCL10 was significantly associated with 25% eGFR decrease [P-value < 0.05, HR 1.733 (1.0076–2.9807)].

Ultimately, after resolution of BKV-DNAemia, acute rejection occurred in 9 patients. No difference was observed between the low-CXCL10 and high-CXCL10 groups (respectively, 13.64 and 16.67%, P > 0.99). However, the time-adjusted uCXCL10 AUC (censored by the rejection date) was higher in the group with subsequent rejection than in the immune-quiescent group [15.5 (11.7) vs 7.49 (6.4) ng/mmol/d, P = 0.05, **Figure 5D**], suggesting a stronger inflammatory response occurs during BKV infection prior to rejection, independent of immunosuppressive regimen weaning (**Figures 4C, D**).

## Discussion

Increased urinary CXCL10 levels have been observed in an increasing number of studies (8, 10–13, 18) in KTRs with BKV reactivation and are usually considered an undesirable confounding factor for the noninvasive diagnosis of acute rejection of the renal allograft. In this study, we reasoned that by revealing the inflammatory status of the graft, uCXCL10 may provide significant and innovative information during the course of BKV reactivation. Therefore, we designed a three-step study to address the diagnostic, prognostic, and predictive performance of uCXCL10 in KTRs with BKV reactivation. Our main results are that (i) uCXCL10 levels gradually increase with urine and blood BKV viral load, but isolated viruria is not a sufficient condition; (ii) uCXCL10 levels are associated with graft function deterioration independent of baseline allograft function, blood viral load or BKVN diagnosis, and subsequent risk of allograft rejection; and (iii) in a longitudinal cohort with serial sampling, uCXCL10 levels at the time of the first BKV-DNAemia identify subsequent inflammatory burden and allograft function.

The cross-sectional cohort in this study reveals the course of uCXCL10 in all stages of BKV replication with a set of three samples (i.e., urine/blood/biopsy) from all patients, including controls. Concordant with our results, Weseslindtner et al. (19) also found that CXCL10 increased in urine in parallel with the gradual development of BKVN. In this study, however, biopsy was performed according to clinical severity, and 27% (15/56) of patients were subsequently classified as having BKVN, while no biopsy was performed for the remaining 73% of KTRs. The rationale for performing biopsy only in the most severe cases with the highest BKV viral load and/or allograft dysfunction is clinically relevant (4), but a consistent pitfall in BKV-related studies is that this approach does not provide an accurate view of the whole spectrum of BKV replication. Our study confirms that urine and blood viral loads are correlated with each other, as previously described (17) and most importantly, with uCXCL10. Here, our systematic approach demonstrates for the first time two key points: isolated viruria does not increase uCXCL10, and BKV-DNAemia increases uCXCL10 similarly whether or not BKVN is diagnosed in the concurrent biopsy.

Consistently, when we investigated eGFR decrease after BKV-DNAemia, histological diagnosis of BKVN was clearly outperformed by uCXCL10 for predicting allograft outcomes. In other words, when a global population is considered, the occurrence of a BKVN is highly likely to be associated with a decline in eGFR. However, when considering a selected population, all with a positive viremia, *i.e.* a probable or presumptive BKVN, then the additional diagnosis of biopsy-proven BKVN does not add much to renal allograft prognosis. BKVN was found in approximately one-third of our BKV-DNAemia cases, emphasizing that BKV damage can be patchy and easily missed by biopsy samples (20) and confirming that sustained BKV-DNAemia may be associated with intragraft damage, whether or not a biopsy has proven it (4, 21). As the intrarenal immune response to control viral replication mediates allograft injury, uCXCL10 might therefore be more relevant than histological evaluation for assessing infection severity in clinical practice.

Of importance, uCXCL10 confounders have been well established. Acute rejection and urinary tract infection were independently associated with increased uCXCL10 levels, whereas proteinuria was not (14). In the present work focusing on all 76 samples with BK viremia, the absence of correlation between uCXCL10 and the urine protein output was confirmed (P = 0.17, **Supplemental Figure 1C**). Finally, uCXCL10 accuracy is minimally influenced by urine concentration or dilution. Hence, uCXCL10 and uCXCL10/creatininuria (*i.e.*, with or without adjusting for urine output volume) exhibit similar diagnostic performances in predicting any type of acute rejection (9).

Another objective here was to describe the natural history of uCXCL10 in the course of BKV infection in an unbiased study. The first longitudinal assessment of urine CXCL10 was published very recently, including 148 samples in 56 KTRs (19). In our longitudinal cohort, all consecutive KTRs at our center were prospectively included (258 patients), and serial measurements of uCXCL10 were performed at predefined time points, resulting in an average of 20 urine samples collected for each patient during the first year post-transplant, providing unprecedented material (1,184 samples from 60 KTRs with BKV-DNAemia) to investigate uCXCL10 during BKV reactivation. Interestingly, in this independent cohort, uCXCL10 at first BKV-DNAemia was predictive of a subsequent inflammatory response trajectory in similar BKV infection at onset, and the uCXCL10/cr threshold was confirmed to be a relevant prognostic biomarker of allograft dysfunction. Finally, integration of the urinary inflammatory burden by computing the area under the uCXCL10/cr curve showed an enhanced inflammatory response in patients with subsequent acute rejection. Overall, high uCXCL10 at first BKV-DNAemia or a high uCXCL10 peak in viremic patients could raise concerns about future allo-immune injury and poor allograft outcomes and necessitate closer medical supervision.

Our results should be considered in light of some limitations. First, the inherent design of the cross-sectional study does not guarantee that the obtained snapshots are representative of all BKV infections. As an example, the median time from BKV infection onset to biopsy was 43 days (**Supplemental Table 4**), but there was a relatively wide range [IQR: 8-121]. Moreover, the relatively low number of patients did not allow us to cover all prognostic markers of graft outcome. Hence, variables that were previously associated with a poor renal outcome (*e.g.*, DSA or ci Banff lesion) failed to reach significance in univariate analysis in this study.

Furthermore, considering the presumed pathophysiology of BKV replication, we expected all patients with detectable viremia to have some degree of urinary viral excretion, and all BKVN to have a detectable DNAemia. However, as illustrated on **Figure 2A**, some discrepant cases were noticed with undetectable BKV viruria in 7 viremic patients and undetectable viremia in 1 BKVN case. For the latter, although this case tested negative twice within 2 weeks before/after the biopsy, high-level BK viremia was diagnosed a month after the biopsy. Moreover, JC virus was *a posteriori* tested positive in the blood, suggesting that both viruses possibly uncounted for the intra-renal lesions and positive SV40 staining. Regarding the other discrepant cases (viremia pos/viruria neg), occurrence of a technical problem during BKV viral load quantification was excluded by performing a second qPCR assay (*i.e.* BKV VP1 mRNA in urine). VP1 was however shown to be the most polymorphic coding region in the BKV genome (22), and variants harboring mismatches in the oligonucleotide binding sites may affect the analytic specificity of established blood assays (23). Although our custom-synthesized primers were designed to primarily target non-polymorphic coding regions, we cannot exclude the possibility that a base change might compromise the binding to the primers. Hence, our sense primer targeted VP1 position from 2,355 to 2,380 while a single-nucleotide polymorphism was subsequently reported at position 2,370 (24).

In addition, the observational nature of the study means that we were unable to prove the benefit of assessing uCXCL10 in KTRs, and only a randomized controlled trial would determine the utility of uCXCL10/cr in the routine clinical work-up. Nevertheless, the longitudinal study of uCXCL10 with innovative trajectory analyses made it possible to identify populations with very different outcomes regarding inflammation and allograft function. The uCXCL10/cr pattern prior to rejection/no rejection will need confirmation in the future, with only 9 rejection episodes in a recent cohort with a short follow-up period. In addition, exclusively imputing this inflammatory response to BKV remains disputable. However, these 60 patients with viremia had a mean 2.4 allograft biopsies/patient from transplantation to last follow-up, of whom a mean of 0.8 biopsy/patient was performed during the BKV replication period. Rejection diagnoses were made in a median time of 180 days after the onset of BK viremia. Altogether, we cannot exclude subclinical rejection episodes occurring between two biopsies; however, the increase in uCXCL10 during the BKV episode as we attempted to describe here is most likely due to BKV replication rather than concurrent rejection.

Finally, the observational nature of the study does not allow us to make conclusions about causality between BKV infection, severity of inflammatory signal in urine, and subsequent rejection episodes, but it paves the way for new research opportunities to understand differential inflammatory responses to BKV infection.

In conclusion, in this study, we specifically addressed the performance of uCXCL10 in the course of BKV reactivation in KTRs. We show that uCXCL10 is not only increased at the time of BKV-DNAemia but also a robust prognostic marker for allograft function. We show that uCXCL10 outperforms many conventional biological and histological parameters, including blood viral load and biopsy-proven BKVN, in predicting the evolution of eGFR. Finally, uCXCL10 is a predictive biomarker, discriminating between different inflammatory responses to BKV infection, with the strongest inflammation eventually leading to eGFR decrease or acute rejection.

## Data Availability Statement

The original contributions presented in the study are included in the article/**Supplementary Material**. Further inquiries can be directed to the corresponding author.

## Ethics Statement

The studies involving human participants were reviewed and approved by Ethics Committee of Ile-de-France XI (#13016). The patients/participants provided their written informed consent to participate in this study.

## Author Contributions

CT and DA conceived and designed the study. CL provided the urine samples and XL collected them. CT, AV, DaP, AD, and LA collected the clinical data, VA-F provided the blood BKV data and RS the DSAs data. CT, AV, VS, and XL carried out the experiments from the cross-sectional study, DoP and SB provided their platform for experiments from the longitudinal study. MR and AV performed the histology reading of biopsies at Necker Hospital. CT and LM performed the statistical analyses and interpreted the data. AS, MT, CL, and FT participated in the interpretation of the results. CT and DA wrote the draft of the report. All authors contributed to the article and approved the submitted version.

## Funding

AD is supported by the National Fund for Scientific Research (Belgium), and AV is supported by the *Société Francophone de Transplantation*. The Emmanuel Boussard Foundation supported DA, LA, LM, CT and VS, and DA received funds from the Day Solvay Foundation.

## Conflict of Interest

The authors declare that the research was conducted in the absence of any commercial or financial relationships that could be construed as a potential conflict of interest.

## Abbreviations

AMR, antibody-mediated rejection; AUC, area under the curve; BKVN, BK virus nephropathy; CI, confidence interval; Cr, urinary creatinine; CFU, colony-forming units; CNI, calcineurin inhibitors; CXCL10, chemokine C-X-C motif ligand 10; D, days; DCD, donation after cardiac death; DSAs, donor-specific antibodies; ECD, expanded-criteria donor; eGFR, estimated glomerular filtration rate; ELISA, enzyme-linked immunosorbent assay; ESRD, end-stage renal disease; GN, glomerulonephritis; IFTA, interstitial fibrosis and tubular atrophy; IQR, interquartile range; KTRs, kidney transplant recipients; Ln, natural logarithm; LOQ, limit of quantification; MDRD, modification of diet in renal disease; NA, not available; NR, no rejection; qPCR, quantitative polymerase chain reaction; RNA, ribonucleic acid; RT, reverse transcription; SCD, standard-criteria donor; SD, standard deviation; TCMR, T cell-mediated rejection; UTI, urinary tract infection.
