# Supplementary material for: Deciphering the Prognostic and Predictive Value of Urinary CXCL10 in Kidney Recipients With BK Virus Reactivation
Source: Front Immunol. 2020 Dec 10;11:604353. doi: 10.3389/fimmu.2020.604353 (PMC7759001; doi:10.3389/fimmu.2020.604353)

Supplemental Appendix for

Deciphering the prognostic and predictive value of urinary CXCL10 in kidney recipients with BK virus reactivation

Table of contents

SUPPLEMENTAL RESULTS ....Page 2
Supplemental Table 1 ....Page 2
Supplemental Table 2 ....Page 3
Supplemental Table 3 ....Page 4
Supplemental Table 4 ....Page 5
Supplemental Table 5 ....Page 7
Supplemental Figure 1 ....Page 8
Supplemental Figure 2 ....Page 9
Supplemental Figure 3 ....Page 10

SUPPLEMENTAL RESULTS

Supplemental Table 1: Oligo sequences and probes used for transcript quantification by qPCR.

Gene	Reference	Sequence	Localization
**BKV VP1**	J02038	Sense: 5'-TGCTGATATTTGTGGCCTGTTTACTA-3'	2355-2380
	K00058	Antisense: 5'-CTCAGGCGGATCTTAAAATATCTTG-3'	2438-2414
	V01108	Probe: 5-FAM-AGCTCTGGAACACAACAGTGGAGAGGCC **TAMRA** 3’	2383-2410
**BAK**	Y13231	Sense: 5' CCCACATCTGGAGCAGAGTCA 3'	192-212
		Antisense: 5' CAGATGCCATTTTTCAGGTCTTG 3'	264-242
		5’ FAM CAGGTGACAAGTGACGGTGGTCTCCA **TAMRA** 3’	215-240

Supplemental Table 2: Patient and transplant characteristics in the cross-sectional study.

Variables	N=391 patients
**Recipient characteristics**	
Men, *n (%)*	241 (61.6)
Age at transplantation (yr), *mean±SD*	47.6±15.7
Cause of ESRD	
GN, *n (%)*	88 (22.5)
Diabetes, *n (%)*	40 (10.2)
Cystic/hereditary/congenital, *n (%)*	91 (23.3)
Secondary GN, *n (%)*	21 (5.4)
Hypertension, *n (%)*	25 (6.4)
Interstitial nephritis, *n (%)*	43 (11.0)
Miscellaneous conditions, *n (%)*	6 (1.5)
Cancer, *n (%)*	0
Etiology uncertain, *n (%)*	77 (19.7)
**Transplant variables**	
Donor age (yr), *mean±SD*[a]	53.8±17.2
Donor type[b]	
Living donor, *n (%)*	107 (27.9)
SCD, *n (%)*	129 (33.6)
ECD, *n (%)*	148 (38.5)
Retransplantation, *n (%)*	83 (21.2)
Delayed graft function, *n (%)*	169 (43.7)
Preformed DSAs, *n (%)*[c]	178 (46.5)
**Immunosuppressive protocol**	
Induction therapy, *n (%)*[d]	369 (98.3)
Basiliximab/Thymoglobuline®, *n (%)*	186 (49.9)/178 (47.7)
Calcineurin inhibitor, *n (%)*[e]	379 (98.2)
Cyclosporin/tacrolimus, *n (%)*	72 (18.7)/307 (79.5)
Purine synthesis inhibitor, *n (%)*[e]	363 (94.0)
Azathioprine/mycophenolic acid, *n (%)*	10 (2.6)/353 (91.5)
Mammalian target of rapamycin inhibitor, *n (%)*[e]	28 (7.3)
Steroids, *n (%)*[e]	383 (99.2)

Abbreviations: DSAs, donor-specific antibodies; ECD, expanded-criteria donor; ESRD, end-stage renal disease; GN, glomerulonephritis; SCD, standard-criteria donor; SD, standard deviation. [a] Data not available (NA) for 3 patients. [b] NA for 7 patients. [c] NA for 8 patients. [d] NA for 18 patients. [e] NA for 5 patients.

Supplemental Table 3: Patient and transplant characteristics in the nested case-control study including 63 single patients with BKV-DNAemia.

**Variables**	**N=63 patients**
**Recipient characteristics**	
Men, *n (%)*	35 (55.6)
Age at transplantation (yr), *mean±SD*	50±17.0
Cause of ESRD	
GN, *n (%)*	15 (23.8)
Diabetes, *n (%)*	7 (11.1)
Cystic/hereditary/congenital, *n (%)*	19 (30.2)
Secondary GN, *n (%)*	2 (3.2)
Hypertension, *n (%)*	5 (7.9)
Interstitial nephritis, *n (%)*	7 (11.1)
Miscellaneous conditions, *n (%)*	0
Cancer, *n (%)*	0
Etiology uncertain, *n (%)*	8 (12.7)
**Transplant variables**	
Donor age (yr), *mean±SD*	58±19.0
Donor type[a]	
Living donor, *n (%)*	16 (26.7)
SCD, *n (%)*	14 (23.3)
ECD, *n (%)*	30 (50.0)
Retransplantation, *n (%)*	10 (15.9)
Delayed graft function, *n (%)*	26 (41.3)
Preformed DSAs, *n (%)*	32 (50.8)
**Immunosuppressive protocol**	
Induction therapy, *n (%)*[b]	59 (100)
Basiliximab/Thymoglobuline®, *n (%)*	24 (40.7)/34 (57.6)
Calcineurin inhibitor, *n (%)*	62 (98.4)
Cyclosporin/tacrolimus, *n (%)*	5 (7.9)/57 (90.5)
Purine synthesis inhibitor, *n (%)*	62 (98.4)
Azathioprine/mycophenolic acid, *n (%)*	1 (1.6)/61 (96.8)
Mammalian target of rapamycin inhibitor, *n (%)*	2 (3.2)
Steroids, *n (%)*	63 (100)

Abbreviations: DSAs, donor-specific antibodies; ECD, expanded-criteria donor; ESRD, end-stage renal disease; GN, glomerulonephritis; SCD, standard-criteria donor; SD, standard deviation. [a] Data not available (NA) for 3 patients. [b] NA for 4 patients

Supplemental Table 4: Sample characteristics in the nested case-control study including 63 single patients with BKV-DNAemia. The low-CXCL10 group was defined as uCXCL10/cr ≤12.86 ng/mmol, and the high-CXCL10 group was defined as uCXCL10/cr >12.86 ng/mmol. MVI is defined by the sum of the glomerulitis and peritubular capillaritis scores.

Variables	All patients (N=63)	Low-CXCL10 group (N=40)	High-CXCL10 group (N=23)	P value
Time from transplantation to biopsy (mo), *median (IQR)*	10 (19.0)	13 (25.5)	6 (8.0)	0.003
**Indication of biopsy**				
Screening biopsy, *n (%)*	4 (6.3)	2 (5.0)	2 (8.7)	0.97
Clinically indicated biopsy, *n (%)*	59 (93.7)	38 (95.0)	21 (91.3)	
Rise in serum creatinine, *n (%)*	23 (39.0)	16 (42.1)	7 (33.3)	0.70
Proteinuria, *n (%)*	0	0	0	
De novo DSAs, *n (%)*	0	0	0	
Control after rejection, *n (%)*	1 (1.7)	0	1 (4.8)	0.76
BKV-DNAemia, *n (%)*	35 (59.3)	22 (57.9)	13 (61.9)	0.98
Other, *n (%)*	0	0	0	
**Banff elementary lesions**				
i score, *mean±SD*[a]	0.21±0.60	0.15±0.54	0.30±0.70	0.14
t score, *mean±SD*	0.90±1.29	0.78±1.21	1.13±1.42	0.37
ci score, *mean±SD*[a]	1.16±1.07	1.21±1.08	1.09±1.08	0.67
ct score, *mean±SD*[a]	1.15±1.05	1.18±1.05	1.09±1.08	0.71
i-IFTA score, *mean±SD*	0.75±1.09	0.80±1.07	0.65±1.15	0.45
ti score, *mean±SD*	0.54±0.96	0.53±0.96	0.57±0.99	0.92
g score, *mean±SD*	0.24±0.64	0.28±0.68	0.17±0.58	0.38
ptc score, *mean±SD*	0.21±0.60	0.10±0.44	0.39±0.78	0.02
MVI score, *mean±SD*	0.44±1.03	0.38±0.90	0.57±1.24	0.56
cg score, *mean±SD*	1.10±0.35	1.10±0.38	1.09±0.29	0.89
ah score, *mean±SD*	2.40±1.06	2.53±1.01	2.17±1.11	0.21
cv score, *mean±SD*[b]	2.57±1.02	2.57±1.01	2.35±1.04	0.35
**Pathologic primary diagnosis**				
Inadequate, *n (%)*	4 (6.3)	2 (5.0)	2 (8.7)	0.97
BKVN, *n (%)*	15 (23.8)	11 (27.5)	4 (17.4)	0.55
Acute rejection, *n (%)*	9 (14.3)	4 (10.0)	5 (21.7)	0.36
Normal, *n (%)*	3 (4.8)	2 (5.0)	1 (4.4)	0.62
Other lesions, *n (%)*[c]	33 (52.4)	22 (55.0)	11 (47.8)	0.77
**BKV infection characteristics**				
Time from 1st blood BKV-positive PCR to biopsy (d), *median (IQR)*	43 (113.0)	45.5 (127.8)	43 (70.5)	0.84

Time from peak DNAemia to biopsy (d), *median (IQR)*	0 (40.5)	0 (44.3)	0 (54.0)	0.20
BKV viral load at biopsy (Log_{10} copies/mL), *median (IQR)*	3.4 (1.8)	3.2 (1.9)	3.4 (1.7)	0.59
Peak BKV viral load (Log_{10} copies/mL), *median (IQR)*	4.6 (1.8)	4.4 (1.8)	4.8 (1.5)	0.16
**Laboratory test results at the time of biopsy**				
Serum creatinine (µmol/L), *mean±SD*	163±51	162±53	165±47	0.67
DSAs, *n (%)*[d]	22 (34.9)	11 (30.6)	11 (47.8)	0.29
Proteinuria/creatininuria ratio (g/g), *mean±SD*	0.38 (0.6)	0.37 (0.6)	0.39 (0.4)	0.19
Urinary CXCL10 (LnCXCL10/cr), *median (IQR)*	2.21 (1.2)	1.94 (1.3)	3.02 (0.8)	<0.0001

Abbreviations: ah, arteriolar hyalinosis; BKVN, BKV-associated nephropathy; cg, allograft glomerulopathy; ci, interstitial fibrosis; ct, tubular atrophy; cv, chronic vascular changes; DSAs, donor-specific antibodies; g, glomerulitis; i, interstitial infiltrate; IFTA, interstitial fibrosis/tubular atrophy; IQR, interquartile range; Ln, natural logarithm; MVI, microvascular inflammation; PCR, polymerase chain reaction; ptc, peritubular capillaritis; SD, standard deviation; t, tubulitis; ti, total inflammation; v, vasculitis. [a] Data not available (NA) for 1 case, [b] NA=8, [c] Including calcineurin inhibitor toxicity, IFTA and recurrent disease, [d] NA=4.

Supplemental Table 5: Patient and transplant characteristics in the longitudinal study including 60 single patients with BKV-DNAemia.

Variables	N=60 patients
**Recipient characteristics**	
Men, *n (%)*	36 (60.0)
Age at transplantation (yr), *mean±SD*	53.6 (15)
Cause of ESRD	
GN, *n (%)*	15 (25.0)
Diabetes, *n (%)*	5 (8.3)
Cystic/hereditary/congenital, *n (%)*	24 (40.0)
Secondary GN, *n (%)*	4 (6.7)
Hypertension, *n (%)*	1 (1.7)
Interstitial nephritis, *n (%)*	2 (3.3)
Miscellaneous conditions, *n (%)*	2 (3.3)
Etiology uncertain, *n (%)*	7 (11.7)
**Transplant variables**	
Donor age (yr), *mean±SD*	56.3 (18)
Donor type	
Living donor, *n (%)*	13 (21.7)
SCD, *n (%)*	16 (26.7)
ECD, *n (%)*	29 (48.3)
DCD, *n (%)*	2 (3.3)
Retransplantation, *n (%)*	17 (28.3)
Delayed graft function, *n (%)*	8 (13.3)
Preformed DSAs, *n (%)*	39 (65.0)
**Immunosuppressive protocol**	
Induction therapy, *n (%)*	60 (100)
Basiliximab/Thymoglobuline®, *n (%)*	22 (36.7)/38 (63.3
Calcineurin inhibitor, *n (%)*	60 (100)
Cyclosporin/tacrolimus, *n (%)*	0 (0)/60 (100)
Mycophenolic acid, *n (%)*	55 (91.7)
Mammalian target of rapamycin inhibitor, *n (%)*	5 (8.3)
Steroids, *n (%)*	60 (100)

Abbreviations: DCD, donation after cardiac death; DSAs, donor-specific antibodies; ECD, expanded-criteria donor; ESRD, end-stage renal disease; GN, glomerulonephritis; SCD, standard-criteria donor; SD, standard deviation.

Supplemental Figure 1: Correlation analyses in the cross-sectional study. (**A**) Correlation between blood and urine BKV viral load. (**B**) Correlation between uCXCL10 and urine BKV viral load in a restricted population after exclusion of possible confounders. (**C**) Correlation between uCXCL10 and urine protein output in all samples with BKV DNAemia. Correlations were computed using Pearson's tests. Abbreviations: UTI, urinary tract infection.

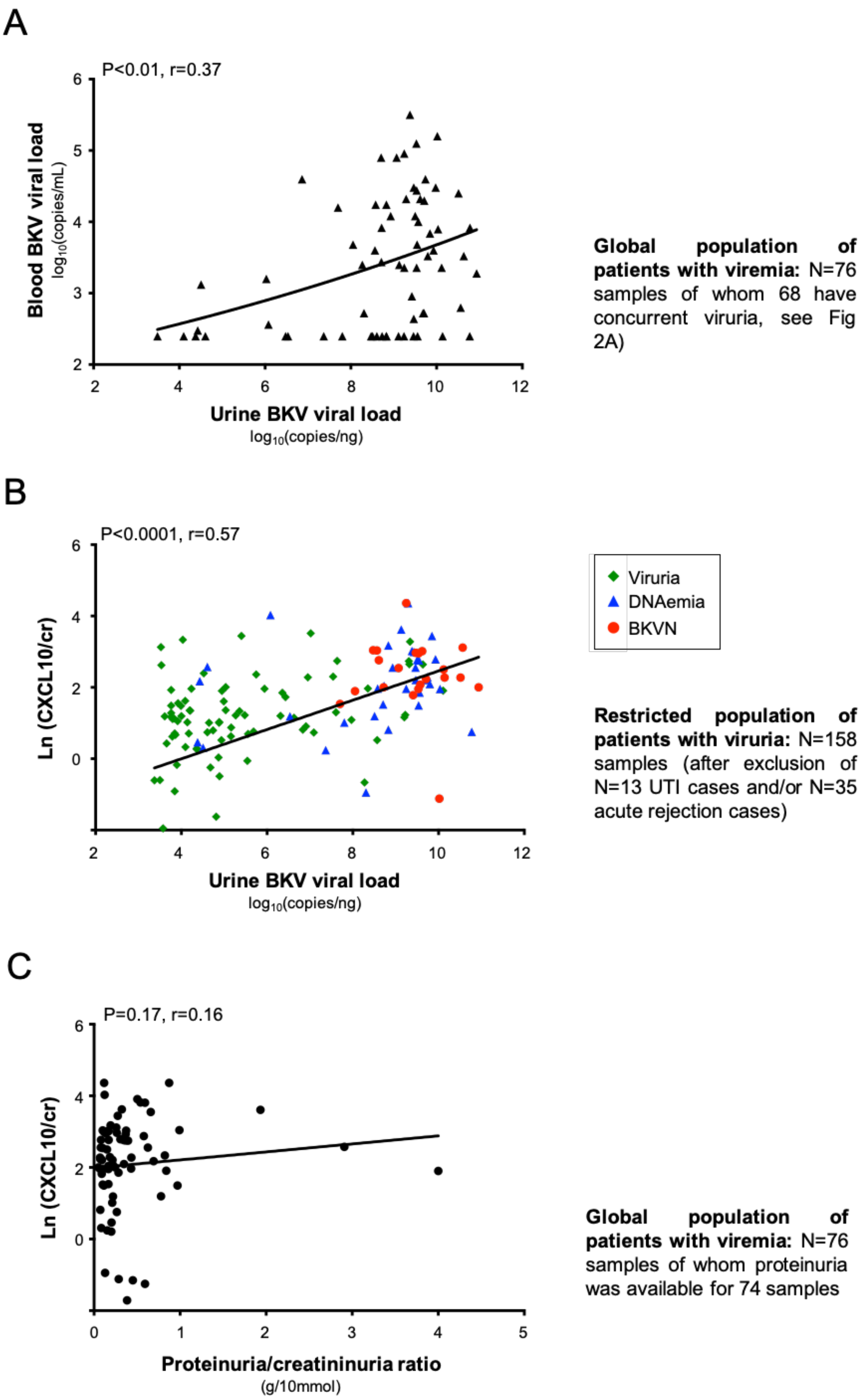

Supplemental Figure 2: Relationship between the rejection phenotypes observed at the time of index biopsy for concurrent BKV-DNAemia and the post-BKV occurrence of rejection in the low-CXCL10 (≤12.86 ng/mmol) and high-CXCL10 (>12.86 ng/mmol) groups from the nested case-control study. Dashed lines indicate a change in category from no rejection to rejection (*i.e., de novo* rejection, in black) or the opposite from rejection to no rejection (in gray). Solid lines indicate patients staying within the same category: with a rejection diagnosis at the time of index biopsy and a post-BKV persistent/recurrent rejection (in black) or no rejection at any time (in gray).

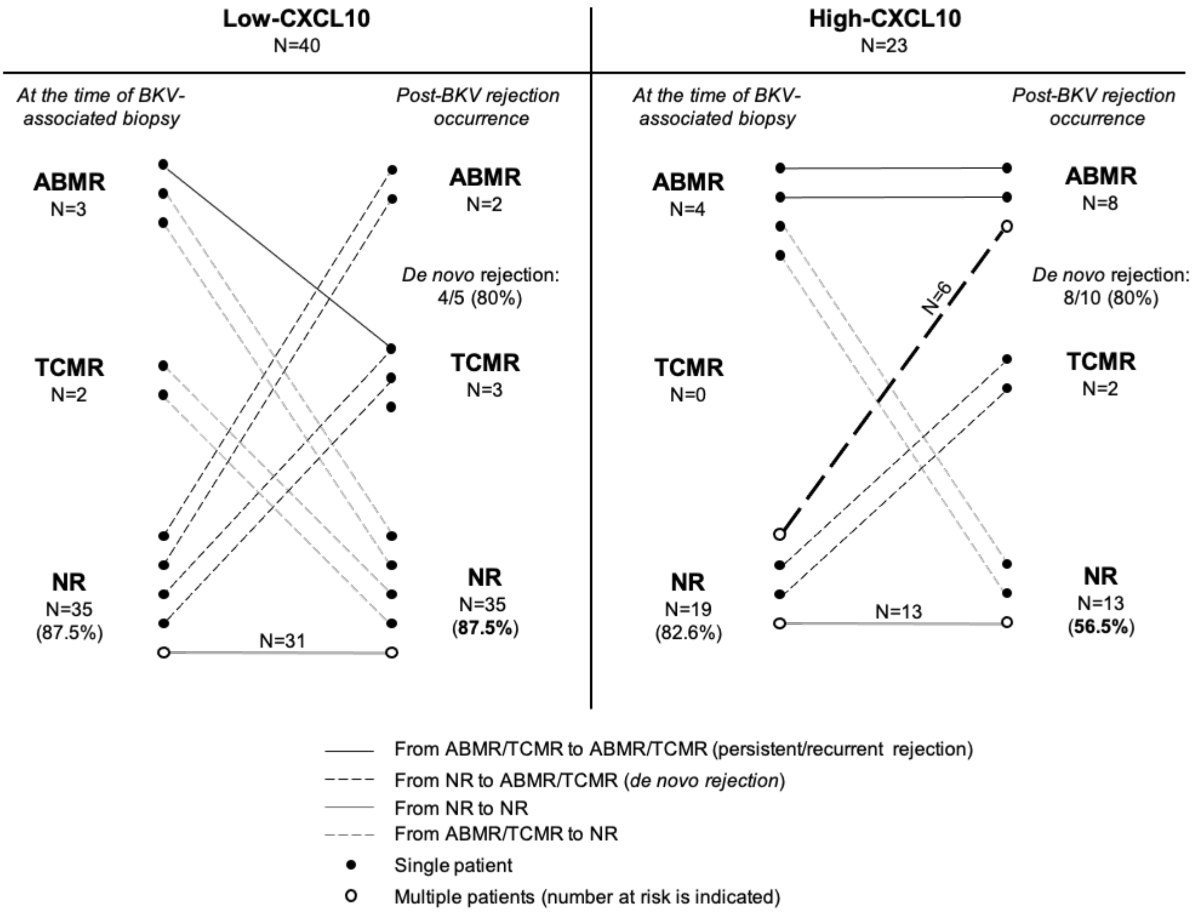

Supplemental Figure 3: Urinary CXCL10/cr (**A**) and blood BKV viral load (**B**) trajectory analyses in the longitudinal cohort split in three groups according to AST Guidelines (Hirsch *et al*, Clin Transplant 2019). Transient BKV-DNAemia is defined by spontaneous BKV clearance without lowering of maintenance immunosuppression (N=18, light gray line). Sustained low-level BKV-DNAemia is defined by peak viremia <4 log_{10}c/mL (N=27 KTRs, dark gray line). Sustained high-level DNAemia is defined by peak viremia ≥4 log_{10}c/mL (N=13 KTRs, black line). Trajectories were computed by regression from longitudinal assessments of uCXCL10/cr (samples collected at biopsy and each outpatient clinic visit during the 1st year post-transplantation) and all available blood BKV viral loads over the same period.

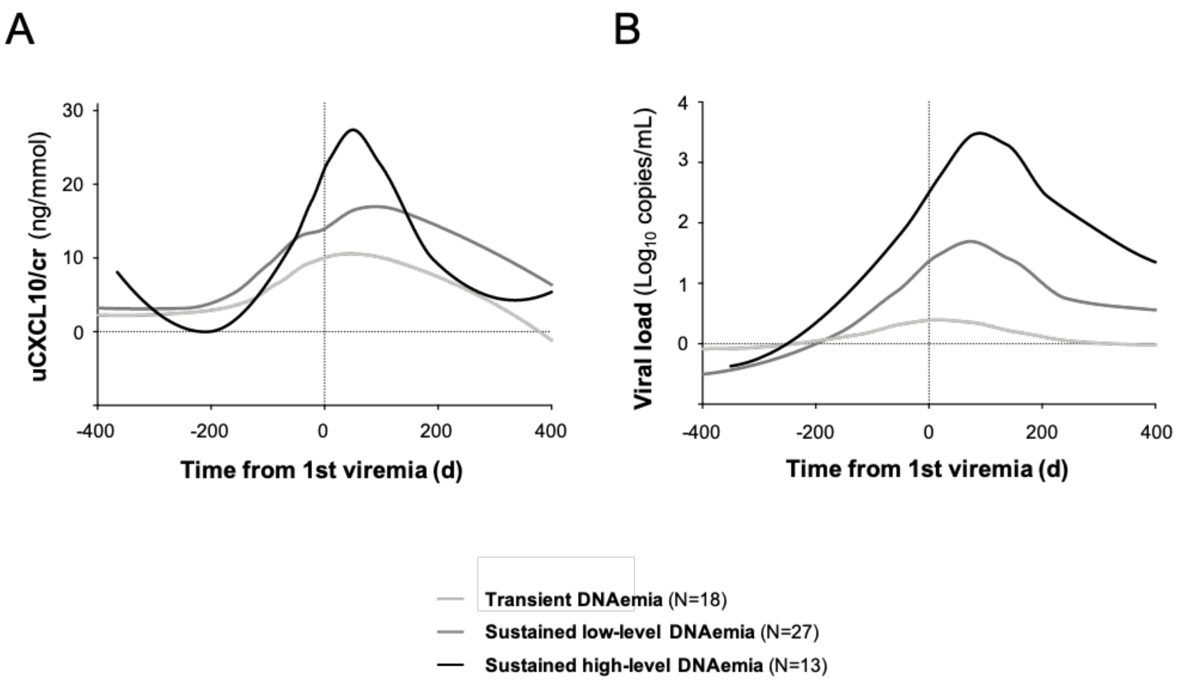

Supplement: Supplementary file 1 [file DataSheet_1.pdf]
